# *Wisteria floribunda* Agglutinin-Positive Mac-2 Binding Protein but not α-Fetoprotein as a Long-Term Hepatocellular Carcinoma Predictor

**DOI:** 10.3390/ijms21103640

**Published:** 2020-05-21

**Authors:** Leona Osawa, Nobuharu Tamaki, Masayuki Kurosaki, Sakura Kirino, Keiya Watakabe, Wan Wang, Mao Okada, Takao Shimizu, Mayu Higuchi, Kenta Takaura, Hitomi Takada, Shun Kaneko, Yutaka Yasui, Kaoru Tsuchiya, Hiroyuki Nakanishi, Jun Itakura, Yuka Takahashi, Nobuyuki Enomoto, Namiki Izumi

**Affiliations:** 1 Department of Gastroenterology and Hepatology, Musashino Red Cross Hospital, Tokyo 180-8610, Japan; r.oosawa@musashino.jrc.or.jp (L.O.); tamaki@musashino.jrc.or.jp (N.T.); kurosaki@musashino.jrc.or.jp (M.K.); sa.kirino@musashino.jrc.or.jp (S.K.); wkkya1234567@gmail.com (K.W.); w.ou@musashino.jrc.or.jp (W.W.); m.okada@musashino.jrc.or.jp (M.O.); drtsss0415@gmail.com (T.S.); mayu.h@musashino.jrc.or.jp (M.H.); tuf029@gmail.com (K.T.); takadahi0107@gmail.com (H.T.); s.kaneko@musashino.jrc.or.jp (S.K.); yutakayas@hotmail.com (Y.Y.); tsuchiya@musashino.jrc.or.jp (K.T.); nakanisi@musashino.jrc.or.jp (H.N.); jitakura@musashino.jrc.or.jp (J.I.); y.takahashi@musashino.jrc.or.jp (Y.T.); 2 First Department of Internal Medicine, Faculty of Medicine, University of Yamanashi, Chuo, Yamanashi 409-3898, Japan; enomoto@yamanashi.ac.jp

**Keywords:** hepatocellular carcinoma, WFA^±^M2BP, AFP, chronic hepatitis C, direct-acting antivirals

## Abstract

Identification of high-risk patients for hepatocellular carcinoma (HCC) after sustained virological responses (SVR) is necessary to define candidates for long-term surveillance. In this study, we examined whether serum markers after 1 year of SVR could predict subsequent HCC development. Total 734 chronic hepatitis C patients without a history of HCC who achieved SVR with direct-acting antivirals were included. The regular surveillance for HCC started from 24 weeks after the end of treatment (SVR24). Factors at SVR24 and 1 year after SVR24 were analyzed for predicting HCC development. During the mean observation period of 19.7 ± 10 months, 24 patients developed HCC. At SVR24, *Wisteria floribunda* agglutinin-positive mac-2 binding protein (WFA±M2BP) ≥ 1.85 and α-fetoprotein (AFP) ≥ 6.0 ng/mL were independent factors of HCC development. However, at 1 year after SVR24, WFA±M2BP ≥ 1.85 was associated with subsequent HCC development (hazard ratio: 23.5, 95% confidence interval: 2.68–205) but not AFP. Among patients with WFA±M2BP ≥ 1.85 at SVR24, 42% had WFA±M2BP < 1.85 at 1 year after SVR24 (WFA±M2BP declined group). Subsequent HCC development was significantly lower in the declined group than in the non-declined group (1 year HCC rate: 0% vs. 9.4%, *p* = 0.04). In conclusion, WFA^±^M2BP but not AFP could identify high and no-risk cases of HCC at 1 year after SVR. Therefore, it was useful as a real-time monitoring tool to identify the candidates for continuous surveillance for HCC.

## 1. Introduction

Direct-acting antivirals (DAAs) treatment for chronic hepatitis C has enabled sustained virological responses (SVR) in several patients in recent years [1,2,3,4,5,6]. However, the number of patients with SVR has increased among the elderly and those with cirrhosis, and the number of patients who develop hepatocellular carcinoma (HCC) after SVR will increase in the future [7]. Therefore, it is clinically important to identify patients at a high risk of developing HCC after SVR and to perform appropriate screening.

Non-invasive fibrosis markers and α-fetoprotein (AFP) levels at 12 or 24 weeks after the end of treatment (SVR12 or 24) are reportedly associated with subsequent HCC development [8,9,10,11,12,13]. *Wisteria floribunda* agglutinin-positive mac-2 binding protein (WFA^±^M2BP) is a novel serum fibrosis marker [14,15], and WFA^±^M2BP at SVR is reported to be associated with HCC development [16,17]. Although WFA^±^M2BP and AFP can identify patients at high risk of HCC development at the time of SVR, it is difficult to continue screening for all cases judged high risk at the time of SVR. Liver fibrosis changes after the achievement of SVR differ from case to case [18], and subsequent changes in fibrosis lead to a different risk of HCC development [19,20]. Therefore, it is necessary to reevaluate the risk of HCC development over time.

Serum markers can be simply and repeatedly measured; therefore, it is considered possible to reevaluate the risk of HCC over time and further narrow down the high-risk cases. However, to our knowledge, no report has examined whether WFA^±^M2BP and AFP at 1 year after SVR are associated with HCC development. Further, it is unclear whether changes in the fibrosis markers alter the risk of carcinogenesis. Therefore, we examined whether WFA^±^M2BP and AFP after 1 year of achieving SVR are associated with HCC development and whether these changes could be used to evaluate the risk of HCC.

## 2. Results

### 2.1. Patient Characteristics

The clinical characteristics and laboratory data of patients are described at the time point of SVR24 and one year after SVR24 in Table 1. Average aspartate aminotransferase (AST) and alanine aminotransferase (ALT) at SVR24 were within the normal ranges because all patients achieved SVR. Twenty-one patients had liver nodules with intermediate probability of HCC (LR3) or probably of HCC (LR4) as defined by the liver imaging reporting and data system (LI-RADS) at entry [21]. There were 501 cases at 1 year after SVR24, excluding 18 patients with HCC development within 1 year. At 1 year after SVR24, AST and ALT were also within the normal ranges. The observation period began at the time of SVR24, and during the mean observation period of 19.7 ± 10 months, 24 patients developed HCC.

#### Association between WFA^±^M2BP and Fibrosis Stage

Association between WFA^±^M2BP and fibrosis stage was examined. Median value of WFA^±^M2BP in F1, F2, F3, and F4 was 0.75, 1.16, 2.06, and 3.01, respectively, and WFA^±^M2BP increased as fibrosis stage increased (*p* < 0.001).

### 2.2. Prediction of HCC Development Using WFA^±^M2BP and AFP at SVR24

**Figure 1:**
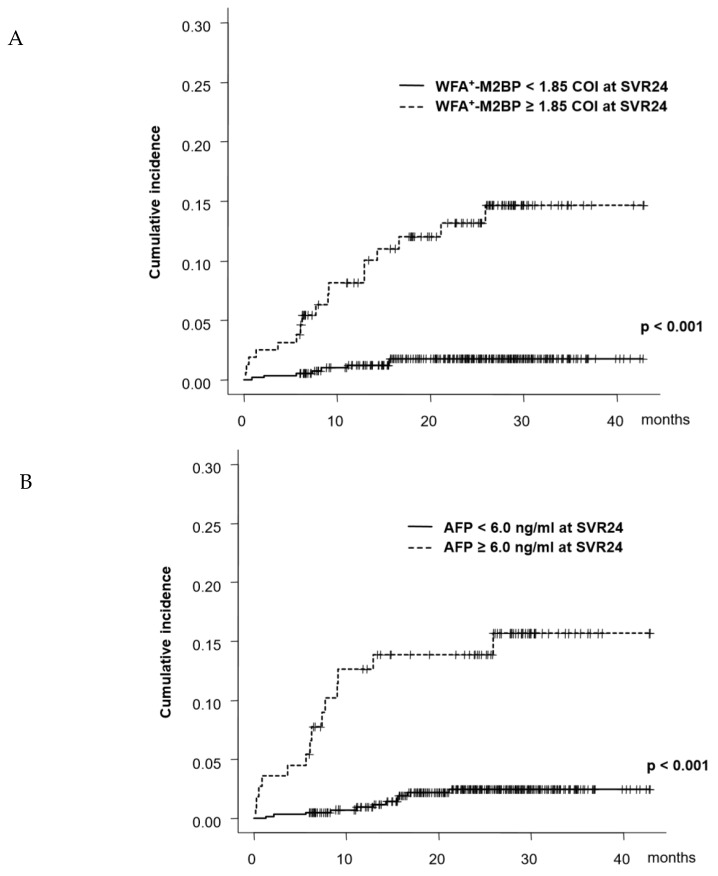
Cumulative incidence of hepatocellular carcinoma (HCC) development sustained virological responses after 24 weeks (SVR24). (**A**) patients were categorized into two groups as per *Wisteria floribunda* agglutinin-positive mac-2 binding protein (WFA^±^M2BP) at SVR24. (**B**) patients were categorized into two groups as per AFP at SVR24.

Serum WFA^±^M2BP and AFP at SVR24 were analyzed for predicting HCC development. ROC analysis was used to select WFA^±^M2BP of 1.85 cut off index (COI) as the optimal cutoff value for predicting HCC development within 1 year. WFA^±^M2BP of <1.85/≥1.85 were defined as low/high risk and low/high-risk patients were 567 (77.2%), and 167 (22.8%), respectively. The AFP level of 6.0 ng/mL was selected as the cutoff value and AFP of <6.0/≥6.0 ng/mL was defined as low/high risk of AFP. The 1-, 2-, and 3-year rate of HCC development in patients with low/high risk of WFA^±^M2BP were 1.2%/1.5%/1.5%, and 8.1%/13.1%/14.6%, respectively. The cumulative rate of HCC development was higher in patients with high risk than those with low risk (*p* < 0.001, Figure 1A). Similarly, the 1-, 2-, and 3-years rates of HCC development in patients with low/high risk of AFP were 0.9%/2.2%/2.2%, and 12.6%/13.8%/15.6%, respectively (Figure 1B). The cumulative rate of HCC development was high in high-risk groups (*p* < 0.001). 

### 2.3. Prediction of HCC Development as Per WFA^±^M2BP and AFP at 1 Year after SVR24

**Figure 2:**
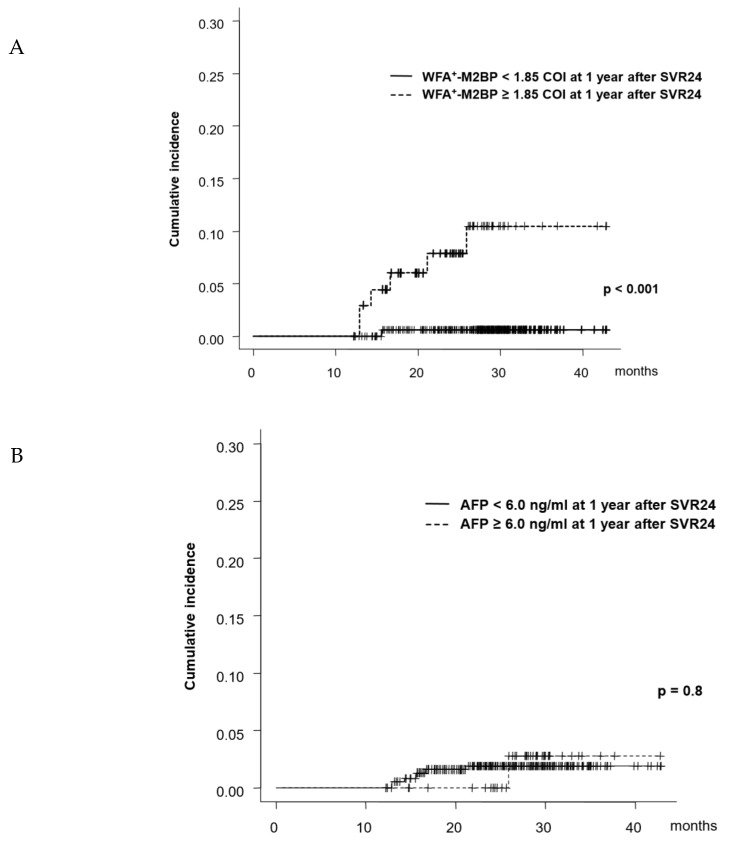
Cumulative incidence of HCC development from 1 year after SVR24 (**A**) patients were categorized into two groups as per the WFA^±^M2BP at 1 year after SVR24. (**B**) patients were categorized into two groups as per the AFP at 1 year after SVR24.

Serum WFA^±^M2BP and AFP at 1 year after SVR24 (78 weeks post-treatment) were analyzed for predicting HCC development thereafter. Using the same cutoff values (WFA^±^M2BP of 1.85 COI and AFP level of 6.0 ng/mL), the cumulative incidence of HCC development was examined. Seven patients developed HCC after 1 year of SVR. The 1- and 2-year rate of HCC development, starting from the time point of 78 weeks post-treatment, in patients with low/high risk of WFA^±^M2BP at 1 year after SVR24 were 0.3%/0.3%, and 8.6%/11.3%, respectively (*p* < 0.001, Figure 2A). In contrast, the 1-, and 2-year rate of HCC development in patients with low/high risk of AFP at 1 year after SVR24 were 1.6%/1.6%, and 0.0%/2.9%, respectively and there was no significant difference between high and low-risk groups (*p* = 0.8, Figure 2B). 

### 2.4. Time-Course Changes in WFA^±^M2BP and AFP and HCC Risk

Time-course changes in WFA^±^M2BP and AFP were examined. WFA^±^M2BP at SVR24 was 1.52 ± 1.4 COI that decreased significantly to 1.28 ± 1.1 COI at 1 year after SVR24 (*p* < 0.001). Similarly, the AFP level at SVR24 was 3.99 ± 3.4 ng/mL and decreased significantly to 3.51 ± 2.2 ng/mL 1 year after SVR24 (*p* < 0.001).

**Figure 3:**
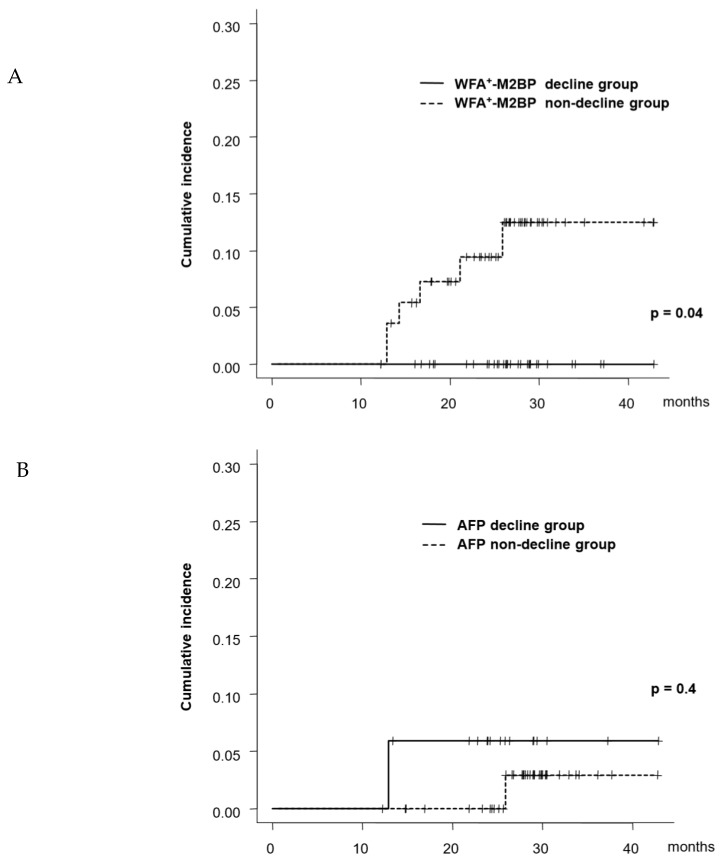
Cumulative incidence of HCC development as per change in the serum marker (**A**) patients were categorized into two groups as per the change in WFA^±^M2BP. Patients with WFA^±^M2BP ≥ 1.85 COI at SVR24 and WFA^±^M2BP < 1.85 at 1 year after SVR24 were defined as the declined group. (**B**) patients were categorized into two groups as per the change in AFP. The patients with AFP ≥ 6.0 ng/mL at SVR24 and AFP < 6.0 at 1 year after SVR24 were defined as the declined group.

Among patients with a high risk of WFA^±^M2BP (≥1.85 COI) at SVR24, WFA^±^M2BP decreased to < 1.85 COI (low risk) at 1 year after SVR24 in 42 patients (42/102, 42%) (WFA^±^M2BP declined group) and remained ≥ 1.85 COI in 60 patients (60%) (WFA^±^M2BP non-declined group). Among patients with a high risk of AFP (≥6.0 ng/mL) at SVR24, AFP decreased to < 6.0 ng/mL at 1 year after SVR24 in 30% (20/67) patients (AFP declined group) and remained ≥ 6.0 ng/mL in 70% of the patients (AFP non-declined group). The 1- and 2-year rates of HCC development, starting from the time point of 78 weeks post-treatment, were 0% and 0%, respectively, in the WFA^±^M2BP declined group, while these rates were 9.4% and 12.4%, respectively, in the WFA^±^M2BP non-declined group. The cumulative incidence of HCC development was significantly higher in patients in the WFA^±^M2BP non-declined group (*p* = 0.04, Figure 3A). In contrast, there was no significant difference in the cumulative rate of HCC development between the AFP declined and non-declined group (Figure 3B). 

### 2.5. Association between AFP and LR3/4 nodule

The association between serum AFP levels and the presence of LR3/4 nodule was analyzed at the time point of SVR24 and 1 year after SVR24. At SVR24, 2.1% (13/623) of patients with AFP at SVR24 < 6.0 ng/m had LR3/4 nodules and 7.2% (8/111) of patients with AFP ≥ 6.0 ng/m had LR3/4 nodules, and presence of LR3/4 nodules was significantly higher in patients with AFP ≥ 6.0 ng/mg (*p* = 0.008). However, at 1 year after SVR24, 0% of the patients with AFP ≥ 6.0 ng/m and 2.1% of those with AFP < 6.0 ng/m had LR3/4 nodules, with no significant difference.

### 2.6. Multivariable Analysis of Factors at SVR24 Associated with HCC Development

Factors at SVR24, including those other than WFA^±^M2BP and AFP, were analyzed for their association with HCC development. Age (every 10 years), albumin, AST (every 30 IU/L), ALT (every 30 IU/L), platelet counts, WFA^±^M2BP ≥ 1.85 COI (hazard ratio [HR]: 9.43, 95% confidence interval (CI): 3.91–22.7, *p* < 0.001, Table 2), AFP ≥ 6.0 ng/mL (HR: 8.17, 95%CI: 2.63–18.4, *p* < 0.001), and presence of LR3/4 nodules (HR: 15.4, 95%CI: 6.06–39.2, *p* < 0.001) were associated with HCC development in the univariate analysis. By using these factors, multivariate analysis revealed that WFA^±^M2BP ≥ 1.85 COI (HR: 5.29, 95% CI: 2.07–13.0, *p* < 0.001), AFP ≥ 6.0 ng/mL (HR: 4.27, 95% CI: 1.84–9.94, *p* < 0.001), and the presence of LR3/4 nodules (HR: 8.49, 95%CI: 3.29–21.9, *p* < 0.001) were independently associated with HCC development.

### 2.7. Multivariable Analysis of Factors at 1 Year after SVR24 Associated with HCC Development

Similarly, factors at 1 year after SVR24, including those other than WFA^±^M2BP and AFP, were analyzed for their association with HCC development. Similar to SVR24, WFA^±^M2BP ≥ 1.85 COI (HR: 35.3, 95% CI: 4.25–293, *p* < 0.001), albumin, platelet counts, and presence of LR3/4 nodules (HR: 60.1, 95%CI: 13.2–273, *p* < 0.001) at 1 year after SVR24 were associated with HCC development in the univariate analysis. However, AFP ≥ 6.0 ng/mL at 1 year after SVR24 was not associated with subsequent HCC development (HR: 1.23, 95% CI: 0.15–10.2, *p* = 0.9). Multivariate analysis revealed that WFA^±^M2BP ≥ 1.85 COI (HR: 23.5, HR: 2.68–205, *p* = 0.004) and presence of LR3/4 nodules (HR:24.1, 95%CI: 5.02–116, *p* < 0.001) were independent factors at 1 year after SVR24 for the prediction of HCC development thereafter.

## 3. Discussion

The present study revealed that WFA^±^M2BP was useful for predicting HCC development at 1 year of SVR; however, AFP was not useful. In addition, even if WFA^±^M2BP was high (≥ 1.85 COI) at SVR, the risk of HCC decreases in patients in whom WFA^±^M2BP subsequently declines (<1.85 COI). However, AFP was useful for predicting HCC at the time of SVR, but not after one year. WFA^±^M2BP could be easily measured and helped identify high cases of HCC development at any time after SVR; therefore, it was useful as a real-time monitor of HCC development risk in the long-term follow-up after SVR.

One of the novel findings of this study was that it confirmed that a high level of WFA^±^M2BP after 1 year of SVR is associated with HCC development risk. The association between WFA^±^M2BP at SVR and HCC risk has been reported by several studies, including our report [16,17]. Since WFA±M2BP at SVR was associated with histological fibrosis stage before DAA treatment, WFA±M2BP at SVR was a risk factor of HCC development after SVR. One advantage of WFA±M2BP is that it can assess the fibrosis stage instead of a liver biopsy and is easy to measure repeatedly. However, to our knowledge, no study has examined the association between WFA^±^M2BP and HCC development risk after long-term follow-up. Early detection of HCC development after DAA treatment requires long-term follow-up. Therefore, not only the prediction of HCC development at SVR time point but also the prediction of HCC development at each follow-up time point is important. We found that WFA^±^M2BP ≥ 1.85 COI at 1 year of SVR, as well as SVR24, were factors for HCC development.

Considering the changes in WFA^±^M2BP after SVR, WFA^±^M2BP generally declines over time. Patients with WFA^±^M2BP ≥ 1.85 COI at SVR were at high-risk for subsequent HCC development; however, those with decreased WFA^±^M2BP after 1 year had a reduced risk of HCC development. However, in some cases, WFA^±^M2BP was still high, and in such cases, the risk of HCC development remained high. The advantage of WFA^±^M2BP is that the change in the HCC risk can be identified by observing the time-dependent changes in WFA^±^M2BP.

Although long-term follow-up is necessary to identify patients with HCC development after SVR, it is difficult to screen all patients frequently. However, the lack of screening after SVR is a known risk factor for the development of advanced HCC [22]. Therefore, there is an urgent need to construct a surveillance strategy after SVR. In surveillance, it is necessary to reevaluate the risk of HCC development not only at SVR but also at follow-up and to narrow down high-risk cases of HCC. WFA^±^M2BP is useful as a real-time monitor for the prediction of HCC development in that WFA^±^M2BP can assess the risk of HCC at any time point and assess changes in HCC risk by observing changes over time; further, clinically important information can be obtained noninvasively and conveniently.

Another novel finding of this study was that AFP after 1 year of SVR was not useful for predicting HCC development. It has been widely reported that AFP levels at SVR are associated with HCC risk [9,10,11,12,17]. This point was also confirmed in this study. However, it became clear that the AFP level 1 year after SVR was not associated with HCC development. It has been reported that high levels of AFP at the time of SVR are associated with the presence of LR3/4 nodules, and such patients are at a high risk of HCC development [23,24]. In this study, high levels of AFP at SVR were associated with the presence of LR3/4 nodules, and the cumulative incidence of HCC development was high in these patients; in particular, many HCC developments were observed within 1 year. Further, the AFP at 1 year after SVR decreased significantly, and no correlation was found when examining the association between AFP and LR3/4 nodules at 1 year after SVR. Although high AFP levels at SVR24 were associated with already existing HCC, high-risk nodules, and early development of HCC, no association between AFP and HCC development after 1 year of SVR, excluding that in these high-risk cases, is believed to exist. AFP was associated with the early development of HCC after SVR; however, one year thereafter, AFP was not useful for predicting HCC development; this was a novel finding of this study. AFP was not useful for predicting HCC development in the long-term follow-up; thus, WFA^±^M2BP, rather than AFP, was useful for HCC monitoring during long-term follow-up after SVR.

There are certain limitations to this study. At present, few cases have been followed up for 2 years. To verify the usefulness of WFA^±^M2BP over time, it is necessary to verify whether WFA^±^M2BP of 2 years after SVR24 and later is useful for prediction of HCC development, which is a future study subject. Moreover, there were few HCC cases; therefore, future long-term studies on a larger cohort are needed to examine the usefulness of WFA^±^M2BP over time. Several fibrosis markers have been confirmed as a predictive marker of HCC development after SVR [13,25]. The diagnostic accuracy of HCC development after SVR should be compared between WFA^±^M2BP and other markers in future studies. 

In conclusion, WFA^±^M2BP but not AFP was useful as a real-time monitor of HCC prediction in long-term follow-up after SVR.

## 4. Materials and Methods

### 4.1. Patients

Between October 2014 and March 2018, 871 patients received DAAs at the Musashino Red Cross Hospital for the treatment of chronic hepatitis C and achieved SVR. Of these, 734 who met the following criteria were enrolled in this study: (1) those who were followed up for 6 months or more after SVR24, (2) had no history of HCC development, and (3) had no co-infection with hepatitis B virus or human immunodeficiency virus. The observation period began at the time of SVR24, and HCC development after SVR was followed up. Written informed consent was obtained from each patient. The study protocol conformed to the ethical guidelines of the Declaration of Helsinki and was approved by the institutional ethics review committee (approval number:28089, 4 April 2017).

### 4.2. Clinical and Laboratory Data

Age and sex of the patients were recorded at SVR24. Fasting blood counts and biochemical tests were also conducted on SVR24 and 1 year after SVR24 using standard methods.

### 4.3. HCC Surveillance and Diagnosis

Ultrasonography and blood tests, including tests for tumor markers, were performed every 3–6 months for HCC surveillance. When tumor marker levels rose abnormally and/or abdominal ultrasonography suggested a lesion suspicious of HCC, contrast-enhanced computed tomography, magnetic resonance imaging, or angiography was performed. HCC was diagnosed for tumors displaying vascular enhancement at the early phase and washout at the later phase as per the guidelines of the American Association for the Study of Liver Diseases, and the Japan Society of Hepatology [26,27]. Tumor biopsy was used to diagnose tumors with non-typical imaging findings.

#### Histological Evaluation

Of all patients enrolled in the study, liver biopsy was performed in 257 patients 1 month prior to the initiation of DAA treatment until the treatment. Liver biopsy specimens were obtained laparoscopically using 13G needles or by percutaneous ultrasound-guided liver biopsy using 15G needles. Specimens were fixed, paraffin-embedded, and stained using hematoxylin–eosin and Masson’s trichrome. A minimum of a 15-mm biopsy sample was required for diagnosis. All liver biopsy samples were independently evaluated by two senior pathologists who were blinded to the clinical data. Fibrosis staging was assessed according to the METAVIR score: F0, no fibrosis; F1, portal fibrosis without septa; F2, portal fibrosis with few septa; F3, numerous septa without cirrhosis; and F4, cirrhosis.

### 4.4. Statistical Analyses

Receiver operating characteristic (ROC) curves and the Youden index were used to determine the optimal cutoff value of WFA^±^M2BP and AFP for predicting HCC development. Statistical significance was defined as a *p*-value < 0.05. Cumulative incidences of HCC development were calculated using the Kaplan–Meier method. The factors associated with HCC development were analyzed using the Cox-proportional hazard model. Correlated factors with a *p*-value < 0.05 in the univariate analysis were used for further multivariate analysis. Backward stepwise selection method was used for multivariate analyses. Association between WFA^±^M2BP and fibrosis stage was analyzed using Spearman’s rank correlation test. Statistical analyses were performed using EZR (Saitama Medical Center, Jichi Medical University, Saitama, Japan) [28] and a graphical user interface for R (The R Foundation for Statistical Computing, Vienna, Austria).

## Figures and Tables

**Table 1:** Patients characteristics.

	At Entry (SVR24)	1 Year after SVR24
	*n* = 734	*n* = 501
Age (years)	65.9 ± 12	67.1 ± 12
Sex (male/female)	291/443	194/307
Albumin (g/dL)	4.32 ± 0.4	4.30 ± 0.4
Bilirubin (mg/dL)	0.67 ± 0.3	0.73 ± 0.3
AST (IU/L)	24.7 ± 8.1	23.7 ± 8.0
ALT (IU/L)	17.6 ± 9.0	17.5 ± 9.0
Platelet counts (×10^4^/μL)	17.0 ± 5.6	17.7 ± 5.6
WFA^±^M2BP (COI)	1.52 ± 1.4	1.28 ± 1.1
AFP (ng/mL)	3.99 ± 3.4	3.51 ± 2.2
Presence of LR3/4 nodules	21 (2.9%)	9 (1.8%)
Histological fibrosis stage (1/2/3/4)	64/75/88/30	

AST, aspartate aminotransferase; ALT, alanine aminotransferase; WFA^±^M2BP, *Wisteria floribunda* agglutinin-positive mac-2 binding protein; COI, cut off index; AFP, alpha fetoprotein; LR, liver imaging reporting and data system; LR3, intermediate probability of HCC; LR4, probably of HCC.

**Table 2:** Factors associated with hepatocellular carcinoma (HCC) development.

	**At SVR24**					
	**Univariate**			**Multivariate**		
	**HR**	**95%CI**	***p* value**	**HR**	**95%CI**	***p* value**
Age (every 10 years)	1.90	1.20–2.99	0.006			
Sex (male)	1.31	0.59–2.93	0.5			
Albumin (g/dL)	0.22	0.09–0.56	0.001			
Bilirubin (mg/dL)	2.34	0.69–7.98	0.2			
AST (every 30 IU/L)	4.40	1.69–11.4	0.002			
ALT (every 30 IU/L)	3.42	1.40–8.35	0.007			
Platelet counts (×10^4^/μL)	0.87	0.80–0.94	<0.001			
WFA^±^M2BP ≥ 1.85 (COI)	9.43	3.91–22.7	<0.001	5.29	2.07–13.0	<0.001
AFP ≥ 6.0 (ng/mL)	8.17	3.63–18.4	<0.001	4.27	1.84–9.94	<0.001
Presence of LR3/4 nodules	15.4	6.06–39.2	<0.001	8.49	3.29–21.9	<0.001
	**1 Year after SVR24**					
	**Univariate**			**Multivariate**		
	**HR**	**95%CI**	***p* value**	**HR**	**95%CI**	***p* value**
Age (every 10 years)	2.40	0.91–6.31	0.08			
Sex (male)	0.64	0.12–3.30	0.6			
Albumin (g/dL)	0.17	0.04–0.65	0.01			
Bilirubin (mg/dL)	2.12	0.27–16.6	0.5			
AST (every 30 IU/L)	2.52	0.46–13.7	0.3			
ALT (every 30 IU/L)	1.18	0.11–12.1	0.9			
Platelet counts (×10^4^/μL)	0.81	0.69–0.94	0.005			
WFA^±^M2BP ≥ 1.85 (COI)	35.3	4.25–293	<0.001	23.5	2.68–205	0.004
AFP ≥ 6.0 (ng/mL)	1.23	0.15–10.2	0.9			
Presence of LR3/4 nodules	60.1	13.2–273	<0.001	24.1	5.02–116	<0.001

HCC, hepatocellular carcinoma; SVR, sustained virological response; HR, hazard ratio; CI, confidence interval, AST, aspartate aminotransferase; ALT, alanine aminotransferase; WFA±M2BP, *Wisteria floribunda* agglutinin-positive mac-2 binding protein; COI, cut off index; AFP, alpha-fetoprotein; LR, liver imaging reporting and data system; LR3, intermediate probability of HCC; LR4, probably of HCC.

## Abbreviations

DAAs	direct-acting antivirals
SVR	sustained virological responses
HCC	hepatocellular carcinoma
AFP	α-fetoprotein
SVR24	24 weeks after the end of treatment
WFA^±^M2BP	*Wisteria floribunda* agglutinin-positive mac-2 binding protein
ROC	receiver operating characteristic
AST	aspartate aminotransferase
ALT	alanine aminotransferase
LR	the liver imaging reporting and data system
HR	hazard ratio
CI	95% confidence interval
